# Efficient full spin–orbit torque switching in a single layer of a perpendicularly magnetized single-crystalline ferromagnet

**DOI:** 10.1038/s41467-019-10553-x

**Published:** 2019-06-13

**Authors:** Miao Jiang, Hirokatsu Asahara, Shoichi Sato, Toshiki Kanaki, Hiroki Yamasaki, Shinobu Ohya, Masaaki Tanaka

**Affiliations:** 1 Department of Electrical Engineering and Information Systems, The University of Tokyo, 7-3-1 Hongo, Bunkyo-ku, Tokyo 113-8656 Japan; 2 Center for Spintronics Research Network (CSRN), Graduate School of Engineering, The University of Tokyo, 7-3-1 Hongo, Bunkyo-ku, Tokyo 113-8656 Japan; 3 Institute of Engineering Innovation, Graduate School of Engineering, The University of Tokyo, 7-3-1 Hongo, Bunkyo-ku, Tokyo 113-8656 Japan

**Keywords:** Spintronics, Two-dimensional materials, Electronic and spintronic devices

## Abstract

Spin–orbit torque (SOT), which is induced by an in-plane electric current via large spin-orbit coupling, enables an innovative method of manipulating the magnetization of ferromagnets by means of current injection. In conventional SOT bilayer systems, the magnetization switching efficiency strongly depends on the interface quality and the strength of the intrinsic spin Hall Effect. Here, we demonstrate highly efficient full SOT switching achieved by applying a current in a single layer of perpendicularly magnetized ferromagnetic semiconductor GaMnAs with an extremely small current density of ∼3.4 × 10^5^ A cm^−2^, which is two orders of magnitude smaller than that needed in typical metal bilayer systems. This low required current density is attributed to the intrinsic bulk inversion asymmetry of GaMnAs as well as its high-quality single crystallinity and large spin polarization. Our findings will contribute to advancements in the electrical control of magnetism and its practical application in semiconductor devices.

## Abstract (web summary)

Spin orbit torque enables an innovative method of manipulating the magnetization of ferromagnets by current injection. Here, Jiang et al. demonstrate efficient full spin–orbit torque switching with an activation current density of ∼3.4 × 10^5^ A cm^−2^ in a single layer ferromagnetic semiconductor GaMnAs.

## Introduction

Spin–orbit torque (SOT) magnetization switching, which is induced by a spin current that is generated by a charge current, is a promising phenomenon that can be used to improve the performance of magnetoresistive random access memory devices. SOT switching allows the read and write paths to be separated and thus enables the independent co-optimization of readability, access latency and energy consumption, thereby decreasing the read error rate. At present, SOT switching has been achieved in metal systems^1–4^ and topological insulator systems^5,6^, which essentially require two functional layers, namely, one ferromagnetic layer and one paramagnetic layer to generate the spin current and inject it into the ferromagnetic layer. The spin current then exerts a torque on the magnetic moment to reverse it^2,7–9^. Hence, the switching efficiency strongly depends on the quality of the interface between the two layers. Furthermore, a large spin polarization in the nonmagnetic layer is necessary for efficient spin injection. Thus, heavy metals and topological insulators with large spin Hall angles, such as Pt, Ta, W, and BiSb^10–12^, are usually used for SOT switching. While topological insulators have been also proposed to achieve an efficient SOT switching using the surface state effect, the switching process is still improvable in terms of the switching hysteresis and its completeness^13^.

**Fig. 1:**
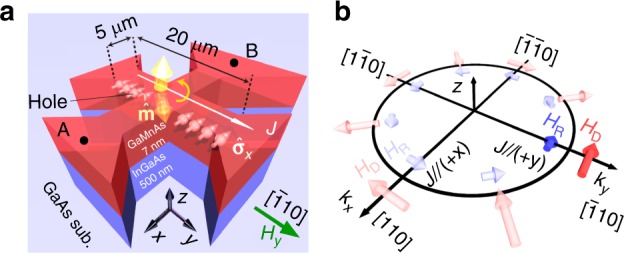
Schematic illustrations of the device structure and the effective magnetic field. **a** Schematic of SOT switching and illustration of the cross-bar device structure of the Ga_0.94_Mn_0.06_As (7 nm)/In_0.3_Ga_0.7_As (500 nm)/GaAs (50 nm) thin film grown on a GaAs (001) substrate. The channel width of the cross-bar is 5 μm. The charge current is applied along the +*y* direction, and the spin component in the −*x* direction (${\hat{\mathbf{\sigma}}}$_*x*_) exerts a torque on the magnetic moment (${\hat{\mathbf{m}}}$) and reverses it. **b** Dresselhaus-like (red) and Rashba-like (blue) effective magnetic fields (*H*_D_ and *H*_R_, respectively) for hole momenta along different crystallographic directions in the tensile Ga_0.94_Mn_0.06_As thin film. (*k*_*x*_, *k*_*y*_) is the wave vector of the holes. The dark colored arrows labeled *H*_D_ and *H*_R_ correspond to the effective magnetic fields when *J* > 0 in the *y* direction

Here, we demonstrate highly efficient full SOT switching that is achieved by applying a current in a single layer of perpendicularly magnetized ferromagnetic semiconductor GaMnAs (Fig. 1a). In a GaMnAs thin film, due to the intrinsic bulk inversion asymmetry of its strained zinc-blende crystal structure and the structural inversion asymmetry induced by the heterostructure, intrinsic spin–orbit interactions couple the spin of a hole with its momentum and generate the effective magnetic field^14–21^. The effective field is contributed by two parts (Fig. 1b), the Dresselhaus-like field (*H*_D_) and Rashba-like field (*H*_R_). Here, the sign and the magnitude of *H*_D_ can be changed by the state of strain^20^. Chernyshov et al. showed partial in-plane magnetization rotation of 90° using the field-like torque in a GaMnAs thin film with in-plane four-fold magnetic anisotropy; however, the prolonged switching process and the efficiency still need to be improved for the realization of full magnetization switching^17^. In this work, we show that the effective fields due to the spin–orbit interactions can induce a spin component whose direction depends on the current orientation and that this spin component can exert a damping-like torque on the magnetic moment, thus enabling efficient 180° magnetization switching with an extremely low current density. In our high-quality single-crystalline GaMnAs thin film, we can expect a low spin-scattering rate, a large effective magnetic field due to the high momentum of the holes originating from impurity-band conduction^22–25^, and a high spin polarization^26^, leading to the successful realization of efficient full SOT switching.

## Results

### Magnetization reversal in the Ga_0.94_Mn_0.06_As single layer

The sample examined in this study is composed of Ga_0.94_Mn_0.06_As (7 nm)/In_0.3_Ga_0.7_As (500 nm)/GaAs (50 nm) grown on a GaAs (001) substrate via molecular beam epitaxy (MBE) (see Supplementary Note 1). The 500-nm In_0.3_Ga_0.7_As layer applies a tensile strain to the Ga_0.94_Mn_0.06_As thin film to induce perpendicular magnetic anisotropy (PMA). The film is patterned into a cross-bar device for transport measurements, as shown in Fig. 1a, with a channel width and length of 5 μm and 20 μm, respectively.

**Fig. 2:**
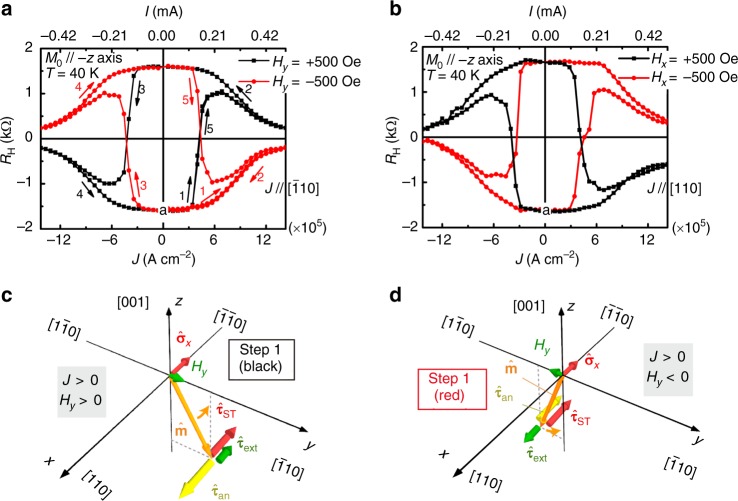
SOT switching (40 K) and the relevant switching mechanism. **a**, **b** Field-assisted SOT switching with *J* // $\left[ {\bar 1{\mathrm{10}}} \right]$ and *H*_*y*_ = ± 500 Oe (**a**) and with *J* // [110] and *H*_*x*_ = ± 500 Oe (**b**). **c**, **d** Illustrations of the torques exerted by the external field (${\hat{\mathbf{\tau }}}_{{\mathrm{ext}}}$), the anisotropy field (${\hat{\mathbf{\tau }}}_{{\mathrm{an}}}$) and the spin component along the –*x* direction $({\hat{\mathbf{\tau }}}_{{\mathrm{ST}}})$ with *J* > 0 when *H*_*y*_ > 0 (**c**) and *H*_*y*_ < 0 (**d**). ${\hat{\mathbf{m}}}$ is in the *y-z* plane. (Source data are provided as a Source Data file)

As shown in Fig. 2a, b, we can successfully achieve current-induced magnetization reversal in the proposed device. We note that the error bar of our experiments is smaller than the size of the data points. Before the transport measurement depicted in Fig. 2a, a large magnetic field of 10 kOe was applied along the –*z* direction to align the initial magnetization *M*_0_ in this direction (point a in Fig. 2a). After decreasing this magnetic field to zero, we applied an external magnetic field *H*_*y*_ along the $[\bar 1{\mathrm{10}}]$ direction (the current direction) to assist in SOT switching and to ensure deterministic magnetization reversal (see Fig. 1a). Then, we measured the Hall resistance (*R*_H_) by measuring the voltage between electrodes A and B shown in Fig. 1a, sweeping a direct current (with a density denoted by *J*) along the *y* direction in the order indicated by steps 1 to 5 on the black curve (*H*_*y*_ = +500 Oe) in Fig. 2a. With this variation of *J*, *R*_H_ varies within approximately ±1.6 kΩ, which is consistent with the *R*_H_ value of the anomalous Hall effect (see Supplementary Note 2 and Supplementary Fig. 2a, i.e., ∼±1.7 kΩ), indicating that the magnetization is fully reversed between the +*z* and −*z* directions by the current. When the current is applied along the $[\bar 1{\mathrm{10}}]$ direction (the +*y* direction), both *H*_D_ and *H*_R_ are generated along the $[\bar 1\bar 10]$ (−*x*) direction (see the dark colored arrows in Fig. 1b). Thus, as shown in Fig. 2c, the hole spin has an *x* component *σ*_*x*_ in the minus direction due to these two effective magnetic fields. ${\hat{\mathbf{\sigma }}}_x$ induces a damping-like torque (DLT) ${\hat{\mathbf{\tau }}}_{{\mathrm{ST}}}$ that is proportional to ${\hat{\mathbf{m}}} \times {\hat{\mathbf{\sigma }}}_x \times {\hat{\mathbf{m}}}$ and whose direction is the same as that of ${\hat{\mathbf{\sigma }}}_x$^2,27^. Here, ${\hat{\mathbf{m}}}$ represents the unit magnetization vector, and ${\hat{\mathbf{\sigma }}}_x$ is the *x* component of the spin polarization vector. In addition to the DLT, there is a torque ${\hat{\mathbf{\tau }}}_{{\mathrm{an}}}$ that is induced by the perpendicular anisotropy field *H*_an_, i.e., ${\hat{\mathbf{\tau }}}_{{\mathrm{an}}} = - {\hat{\mathbf{m}}} \times {\hat{\mathbf{H}}}_{{\mathrm{an}}}$, and a torque ${\hat{\mathbf{\tau }}}_{{\mathrm{ext}}}$ that is induced by *H*_*y*_, i.e., ${\hat{\mathbf{\tau }}}_{{\mathrm{ext}}} = - {\hat{\mathbf{m}}} \times {\hat{\mathbf{H}}}_y$. When *H*_*y*_ = +500 Oe, in the initial magnetization state *M*_0_ along the −*z* direction, the magnetization is slightly tilted towards the +*y* direction. In this case, when *J* > 0, ${\hat{\mathbf{\tau }}}_{{\mathrm{ST}}}$ points in the same direction as ${\hat{\mathbf{\tau }}}_{{\mathrm{ext}}}$, which is opposite to the direction of ${\hat{\mathbf{\tau }}}_{{\mathrm{an}}}$ (Fig. 2c). With increasing *J*, ${\hat{\mathbf{\tau }}}_{{\mathrm{ST}}}$ is enhanced and reverses the magnetic moment with the assistance of ${\hat{\mathbf{\tau }}}_{{\mathrm{ext}}}$ (step 1 on the black curve in Fig. 2a). Subsequently, as *J* decreases to 0, ${\hat{\mathbf{\tau }}}_{{\mathrm{ST}}}$ becomes 0, and the magnetic moment then points in the +*z* direction (step 2 on the black curve). Similarly, when *J* < 0, the magnetic moment is pulled back to the −*z* direction via the *y* > 0 side (steps 3 and 4 on the black curve), where the directions of ${\hat{\mathbf{\tau }}}_{{\mathrm{ST}}}$, ${\hat{\mathbf{\tau }}}_{{\mathrm{an}}}$ and ${\hat{\mathbf{\tau }}}_{{\mathrm{ext}}}$ are the opposite of what they are in the abovementioned case of *J* > 0. Finally, as *J* again increases in the positive direction (step 5 on the black curve), the switching process occurs in the same way as in step 1 on the black curve, which indicates that the current-induced switching process is repeatable. By contrast, when *H*_*y*_ = −500 Oe (see the illustrations of the torques in Fig. 2d), the switching polarity can be symmetrically changed with the reversal of the sign of *H*_*y*_ (see the black and red curves in Fig. 2a), which is a typical characteristic of SOT switching in PMA thin films^7^. Here, the critical switching current density *J*_c_ is 3.43 × 10^5^ A cm^−2^ with *H*_*y*_ = 500 Oe at 40 K. This value of *J*_c_ is two orders of magnitude less than that in metal systems, which is usually on the order of 10^7^ A cm^–2 ^^1,7,28^.

We can see that by changing the directions of *J* and the external magnetic field from $[\bar 110]$ to [110] (i.e., *H*_*x*_), the *H*_D_ is dominant whereas the *H*_R_ is negligibly small in our GaMnAs film, as shown below. In this case, when *J* > 0 (in the +*x* direction), *H*_D_ is generated along $[1\bar 10]$, but *H*_R_ is along $[\bar 110]$, as shown in Fig. 1b. As shown in Fig. 2b, the current-induced SOT switching curves with an *H*_*x*_ of ±500 Oe, which start with the initial magnetization state in the −*z* direction (point a), present the opposite polarities relative to the results in Fig. 2a. This finding indicates that the direction of the total effective magnetic field relative to the current direction in Fig. 2b is different from that in Fig. 2a. As shown in Fig. 1b, while the direction of *H*_R_ relative to *J* [//(+*x*)] is the same as that when *J* // (+*y*), the relative direction of *H*_D_ is the opposite. Thus, *H*_D_ is dominant in our system. Furthermore, based on the nearly identical values of *J*_c_ between Fig. 2a, b, we can conclude that *H*_R_ is negligibly small. Because the Rashba effect is relevant only near the interfaces, whereas the current flows mainly in the bulk of the GaMnAs layer in our study, this conclusion is reasonable.

### Estimation of the heating effect and switching phase diagram

**Fig. 3:**
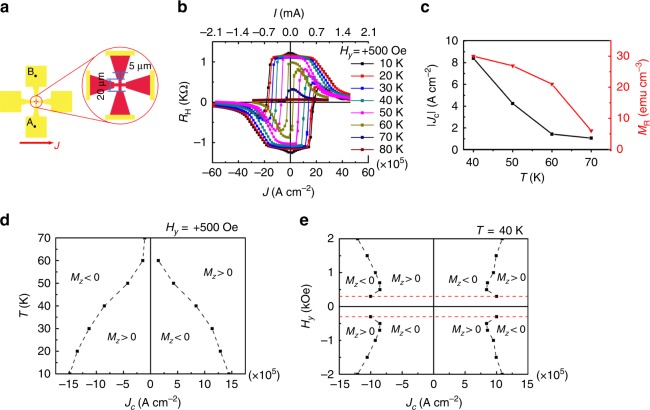
Estimation of the heating effect and switching phase diagrams. **a** Schematic illustration of the device with Au (100 nm)/Cr (5 nm) electrodes as heat sinks. **b** SOT switching at various temperatures. **c** Temperature dependences of *J*_c_ with an external magnetic field (*H*_*y*_) of 500 Oe applied along the $[\bar 1{\mathrm{10}}]$ axis and remanent magnetization *M*_R_. **d**, **e** Switching phase diagram of the Ga_0.94_Mn_0.06_As thin film about temperature (*T*) (**d**) and applied external magnetic field (*H*_*y*_) along the $[\bar 1{\mathrm{10}}]$ axis (**e**). (Source data are provided as a Source Data file)

As shown in Fig. 3a, to estimate the heating effect during the measurement shown in Fig. 2a, b, we made another cross-bar device with electrodes covered with Au (100 nm)/Cr (5 nm) as heat sinks (see the yellow parts in Fig. 3a), using the same MBE-grown wafer. The temperature (*T*) dependence of the SOT switching in this device is shown in Fig. 3b, indicating that the magnetization switching process is basically stable at *T* up to 60 K. As shown in Fig. 3c, both *J*_c_ and the remanent magnetization (*M*_R_) nearly disappear at *T* = 70 K. Hence, we conclude that the heating effect is negligibly small in this device with metal electrodes.

We summarize the switching behavior shown in Fig. 3b as a phase diagram in Fig. 3d, which indicates that *J*_c_ decreases with the increase of *T*. Combining these results with the magnetization hysteresis curves of the Ga_0.94_Mn_0.06_As thin film measured with various magnetic field orientations (see Supplementary Fig. 3), the decrease of *J*_c_ can be attributed to the decrease in the anisotropy field. The actual temperature during the measurement shown in Fig. 2a, b might be around 10 K higher than the set value (*T*_set_ = 40 K) because the critical switching current density of 3.4 × 10^5^ A cm^−2^ is closed to the value of 4.2 × 10^5^ A cm^−2^ obtained at *T* = 50 K in the device with the Au/Cr capping layer. This estimation is also consistent with the fact that the shape of the switching curve is firmly square-like in Fig. 2a, b at *T*_set_ = 40 K like the ones obtained at *T* below 50 K in the device with the Au/Cr capping layer (Fig. 3b). In addition, we carried out measurements of SOT switching with various external magnetic fields *H*_*y*_ applied along the *y* direction and summarized them as a switching phase diagram as shown in Fig. 3e, indicating that *J*_c_ decreases with the increase of *H*_*y*_ when *H*_*y*_ is lower than 500 Oe but increases with the increase in *H*_*y*_ when *H*_*y*_ is larger than 500 Oe. When *H*_*y*_ is lower than 500 Oe, *H*_*y*_ assists the SOT switching and decreases the switching current density, which is consistent with the typical SOT switching. However, the origin of the behavior when *H*_*y*_ is larger than 500 Oe is unclear at present and needs further studies. *H*_*y*_ may tilt part of the magnetization along this magnetic-field direction, and the magnetization switching may also occur near the film plane^17^. In this case, large *H*_*y*_ does not aid the switching process but hinders it, resulting in the increase of the switching current density.

### Spin–orbit torque strength in the Ga_0.94_Mn_0.06_As thin film

**Fig. 4:**
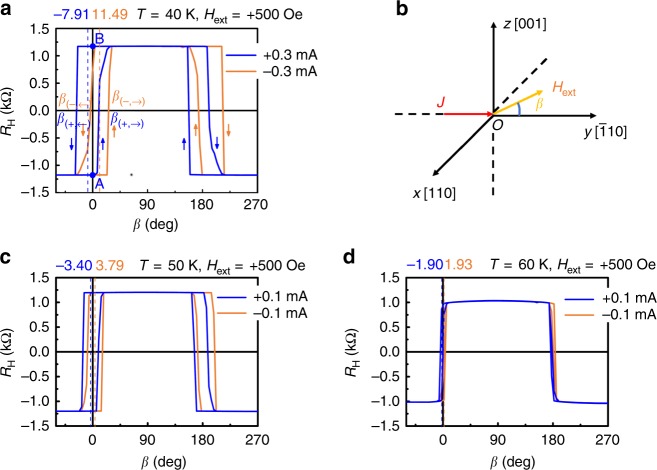
Spin–orbit torque strength in the Ga_0.94_Mn_0.06_As thin film. **a** *R*_H_ measured with a current of ±0.3 mA and an external field of 500 Oe applied at an angle *β* from the $\left[ {\bar 1{\mathrm{10}}} \right]$ direction in the *y-z* plane. The arrows in the graph represent the sweeping directions of *β*. **b** Illustrations of the current (*J*), the external magnetic field (*H*_ext_) and *β*. **c**, **d** *R*_H_ measured with the current of ±0.1 mA and an external field of 500 Oe applied at an angle *β* from the $\left[ {\bar 1{\mathrm{10}}} \right]$ direction in the *y-z* plane at *T* = 50 K (**c**) and *T* = 60 K (**d**). (Source data are provided as a Source Data file)

The SOT strength can be quantitatively characterized by the equivalent magnetic field (*H*_equi_)^29^. We measured *R*_H_ at 40 K with a current of ±0.3 mA applied along the $[\bar 1{\mathrm{10}}]$ direction and a fixed external magnetic field of 500 Oe applied at an angle *β* from the $[\bar 1{\mathrm{10}}]$ direction in the *y-z* plane as shown in Fig. 4a, b. When the current is positive (see the blue lines in Fig. 4a), ${\hat{\mathbf{\tau }}}_{{\mathrm{ST}}}$ is along the –*x* direction, which gives a force to the magnetization in the counterclockwise direction when we see it from the +*x* direction (see Fig. 2c). At point A in Fig. 4a with the magnetization in the –*z* direction, the increase in *β* helps the magnetization rotate in this counterclockwise direction, and thus the magnetization can easily rotate. However, at point B in Fig. 4a with the magnetization in the +*z* direction, because the magnetic field direction is the opposite to the rotation direction of the magnetization, it does not rotate until the –*z* component of the magnetic field becomes large when *β* is increased in the negative direction. Therefore, one can see that the asymmetry of the data around *β* = 0 between the different sweep directions of *β* in Fig. 4 is related to the SOT. When the angle *β* is small, the effective magnetic field *H*_equi_ that is equivalent to the SOT strength is given by *H*_ext_·*β*_av_^29^. Here, *β*_av_ is the average of the *β* values at the magnetization switching with the different sweep directions of *β* for positive and negative current directions: *β*_av_ = (|*β*_(*+*, *→*)_ + *β*_(*+*, *←*)_| + *|β*_(*−*, *→*)_ + *β*_(*−*, *←*)_*|*)/4, where the subscript arrow represents the sweeping direction of *β*, and ± represent the current direction. Here, *β*_av_ is estimated to be (7.91° + 11.49°)/2 = 9.7°. Thus, *H*_equi_ is estimated to be 500 × 9.7 × π/180 = 84.6 Oe and the efficiency of the equivalent field, *χ* = *H*_equi_/*J*_c_, where *J*_c_ is 8.5 × 10^5^ A cm^−2^ at 40 K in Fig. 3b, is estimated to be 99 Oe/(10^6^ A cm^−2^), which is almost two orders of magnitude lager than that [1.7 Oe/(10^6^ A cm^−2^)] in the Pt/Co bilayer system, indicating that very efficient magnetization switching is realized in GaMnAs. Figure 4c, d shows the SOT strength measured at *T* = 50 K and *T* = 60 K with a current of *I* = ±0.1 mA (less than the switching current), from which *H*_equi_ is estimated to be 31 Oe at *T* = 50 K and 17 Oe at *T* = 60 K. Hence, we can conclude that the SOT strength decreases with the increase of temperature because the effective field becomes weak.

### Landau–Lifshitz–Gilbert (LLG) simulation

The obtained *R*_H_ − *J* curves are well reproduced by the LLG equation, which confirms the scenario described above. Here, we consider the SOT switching result obtained at 40 K for the device with metal electrodes as heat sinks (see Fig. 3b) (i.e., *J* // *y* with *H*_*y*_). The LLG equation is expressed as

1 $$\dot{{\widehat{\mathbf{m}}}} = - \gamma \,{\hat{\mathbf{m}}} \times {\hat{\mathbf{H}}} + \alpha \,{\hat{\mathbf{m}}} \times {\dot{\hat{\mathbf{m}}}} + \zeta _{{\mathrm{DLT}}}\left( {{\hat{\mathbf{m}}} \times {\hat{\mathbf{\sigma }}}_x \times {\hat{\mathbf{m}}}} \right) + \zeta _{{\mathrm{FLT}}}\left( {\hat{\mathbf{\sigma }}}_x \times {\hat{\mathbf{m}}} \right),$$

where $\dot{{\widehat{\bf{m}}}}$ is the derivative of ${\hat{\bf{m}}}$ with respect to time; ${\hat{\mathbf{H}}}$ is the effective field consisting of the external field, the anisotropy field, *H*_D_ and *H*_R_; *γ* is the gyromagnetic ratio; *α* is the damping constant; $\zeta _{{\mathrm{DLT}}}$ is the DLT coefficient; and $\zeta _{{\mathrm{FLT}}}$ is the field-like torque (FLT) coefficient. Here, we replace $\zeta _{{\mathrm{DLT}}}{\hat{\mathbf{\sigma }}}_x$ with ${\it{r\gamma }}{\hat{\mathbf{S}}}_x$ and $\zeta _{{\mathrm{FLT}}}{\hat{\bf{\sigma }}}_x$ with $\frac{{\gamma (1 - r)}}{\alpha }{\hat{\bf{S}}}_x$, where ${\hat{\bf{S}}}_x$ is the effective magnetic field in the *x* direction and *r* expresses the strength of the DLT relative to the total SOT: when *r* is 0, only the FLT is present, and when *r* is 1, only the DLT is present. As shown in Supplementary Note 4, the LLG equation can be transformed into

2 $$\frac{{{\dot{\hat{\bf{m}}}}}}{{\gamma {\prime}}} = - {\hat{\bf{m}}} \times ({\hat{\mathbf{H}}} + \beta {\hat{\bf{S}}}_x) - \alpha {\hat{\bf{m}}} \times {\hat{\bf{m}}} \times \left( {{\hat{\bf{H}}} + \frac{1}{\alpha }{\hat{\bf{S}}}_x} \right),$$

where

3 $$\gamma {\prime} = \frac{\gamma }{{1 + \alpha ^2}},\,\beta = \frac{{1 - r({\mathrm{1}} + \alpha ^2)}}{\alpha }.$$

**Fig. 5:**
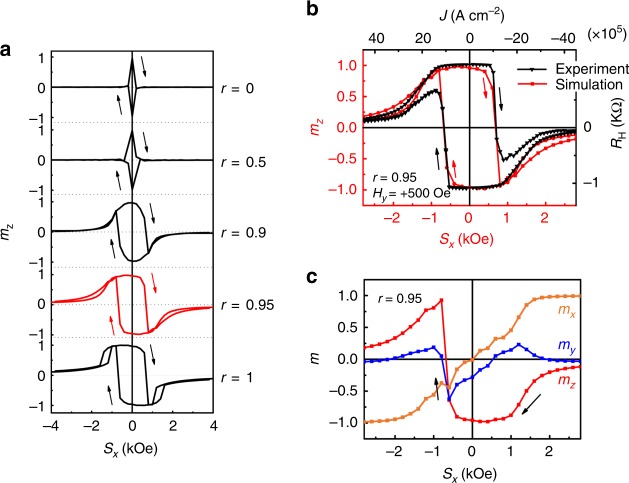
LLG simulation results and comparison with experimental results. **a** Simulated SOT switching with different *r* values of 0, 0.5, 0.9, 0.95 and 1. The parameters used here are *H*_*y*_ = 500 Oe, *H*_an_ = 2.13 kOe and *α* = 0.05. **b** Simulated SOT switching at *r* = 0.95 and experimental results with *H*_*y*_ = 500 Oe. Note that the direction of the *J* axis is reversed because the signs of *J* and *S*_*x*_ are opposite [e.g., when *J* > 0, *S*_*x*_ (or *σ*_*x*_) < 0, as shown in Fig. 2c]. **c** Calculated values of *m*_*x*_, *m*_*y*_ and *m*_*z*_ as functions of *S*_*x*_. (Source data are provided as a Source Data file)

When *H*_*y*_ = 500 Oe, *H*_an_ = 2.13 kOe (for the estimation of *H*_an_, see Supplementary Note 2), and *α* = 0.05^30^, by solving the LLG equation for various *r* values, we obtained the quasi-static magnetization state in which ${\dot{\hat{\bf{m}}}}$ becomes zero for various *S*_*x*_ as shown in Fig. 5a. When *r* is 0, only the FLT is present, and no switching occurs. With increasing *r*, the switching curve shows characteristics much more similar to those of the experimental results, especially when *r* is 0.95, as shown in Fig. 5b, indicating that both the DLT and FLT act on SOT switching but that the DLT is dominant. Note that the direction of the *J* axis in Fig. 5b is reversed because the signs of *J* and *S*_*x*_ are opposite [e.g., when *J* > 0, *S*_*x*_ (or *σ*_*x*_) < 0, as shown in Fig. 2c]. Figure 5c shows the calculated values of *m*_*x*_, *m*_*y*_ and *m*_*z*_, which are the *x*, *y* and *z* components of ${\hat{\mathbf{m}}}$, respectively, as functions of *S*_*x*_. After the magnetic moment rotates to the opposite direction at *S*_*x*_ = −0.8 kOe, *m*_*y*_ and *m*_*z*_ become close to 0 and *m*_*x*_ becomes close to –1 as *S*_*x*_ increases to −2.4 kOe, indicating that the magnetic moment ultimately points along the −*x* axis (in the same direction as the DLT).

## Discussion

In this work, we have found that SOT switching can be achieved in a single-crystalline ferromagnet with intrinsic bulk inversion asymmetry, strong spin–orbit interactions and a large spin polarization. Furthermore, the switching current density *J*_c_ is very low (3.43 × 10^5^ A cm^−2^) because of the large effective magnetic field, which is expected due to the large momentum of the holes originating from impurity-band conduction^22–25^. Our results provide us with guidance in selecting appropriate materials and offer a new possibility for achieving more efficient electrical control of magnetism, which will facilitate the development of SOT switching devices for practical applications.

## Methods

### Sample preparation

The Ga_0.94_Mn_0.06_As thin film was grown on a semi-insulating GaAs (001) substrate in an ultrahigh-vacuum MBE system. After the removal of the surface oxide layer of the GaAs substrate at 580 °C, a 50-nm-thick GaAs buffer layer was grown to obtain an atomically smooth surface. After that, the substrate was cooled down to 450 °C for the growth of In_0.3_Ga_0.7_As with a thickness of 500 nm to induce a tensile strain on the Ga_0.94_Mn_0.06_As layer, giving rise to PMA. Then, the sample was cooled down to 243 °C for the growth of the 7-nm Ga_0.94_Mn_0.06_As layer. The growth process was monitored in situ by means of reflection high-energy electron diffraction. The Curie temperature of the Ga_0.94_Mn_0.06_As layer was estimated to be 88 K (see Supplementary Note 1).

### Device preparation and electrical measurements

The sample was patterned into a cross-bar device with a width of 5 μm using photolithography and argon ion milling. For the SOT measurements, a Keithley 2636 A instrument was used as the current source for applying the direct current. The Hall voltage was measured with a Keithley 2400 apparatus. The measurements were carried out at 40 K.

## Supplementary information

Supplementary Information

Peer Review File

Source Data

## Data Availability

The datasets analyzed during this study are available at the 4TU. ResearchData repository, 10.4121/uuid:ede92a7d-6b44-4dcd-b555-7d9f76993dcc (ref. ^31^).
