# Supplementary material for: Efficient full spin–orbit torque switching in a single layer of a perpendicularly magnetized single-crystalline ferromagnet
Source: Nat Commun. 2019 Jun 13;10:2590. doi: 10.1038/s41467-019-10553-x (PMC6565668; doi:10.1038/s41467-019-10553-x)
Supplement: Supplementary file 1 — Supplementary Information [file 41467_2019_10553_MOESM1_ESM.pdf]

Supplementary Information

Efficient full spin-orbit torque switching in a single layer of a perpendicularly magnetized single-crystalline ferromagnet

Miao Jiang[1*], Hirokatsu Asahara[1], Shoichi Sato[1], Toshiki Kanaki[1], Hiroki Yamasaki[1], Shinobu Ohya[1,2,3*] and Masaaki Tanaka[1,2*]

[1]*Department of Electrical Engineering and Information Systems, The University of Tokyo, 7-3-1 Hongo, Bunkyo-ku, Tokyo 113-8656, Japan*

[2]*Center for Spintronics Research Network (CSRN), Graduate School of Engineering, The University of Tokyo, 7-3-1 Hongo, Bunkyo-ku, Tokyo 113-8656, Japan*

[3]*Institute of Engineering Innovation, Graduate School of Engineering, The University of Tokyo, 7-3-1 Hongo, Bunkyo-ku, Tokyo 113-8656, Japan*

[*]E-mail: miao@cryst.t.u-tokyo.ac.jp; ohya@cryst.t.u-tokyo.ac.jp; masaaki@ee.t.u-tokyo.ac.jp

Supplementary Note 1. Fundamental properties of the $Ga_{0.94}Mn_{0.06}As$ thin film

We used in-situ reflection high-energy electron diffraction (RHEED) to monitor the crystallinity during the growth. The diffraction pattern of the $Ga_{0.94}Mn_{0.06}As$ layer was 1×2 as shown in Supplementary Fig. 1a, the streaky pattern of which indicates that a high-quality zinc-blende-type single-crystal structure was obtained. Supplementary Figure 1b shows the transmission electron microscopy (TEM) lattice image of the $Ga_{0.94}Mn_{0.06}As/In_{0.3}Ga_{0.7}As$ thin film, which shows that the single crystalline $Ga_{0.94}Mn_{0.06}As$ layer is epitaxially grown on the $In_{0.3}Ga_{0.7}As$ buffer layer. The perpendicular-magnetic-field dependence of the magnetic circular dichroism (MCD) of the $Ga_{0.94}Mn_{0.06}As$ thin film at 5 K is shown in Supplementary Fig. 1c. With the square-like curve, we can confirm that the $Ga_{0.94}Mn_{0.06}As$ thin film shows perpendicular magnetic anisotropy (PMA) induced by the tensile strain applied by the 500-nm $In_{0.3}Ga_{0.7}As$ layer underneath the $Ga_{0.94}Mn_{0.06}As$ layer. In addition, the Curie temperature (T_C) of the $Ga_{0.94}Mn_{0.06}As$ thin film is estimated to be around 88 K by the measurements of saturation magnetization M_s *vs.* temperature with a magnetic field H_z applied perpendicular to the film plane using a superconducting quantum interference device (SQUID) as shown in Supplementary Fig. 1d.

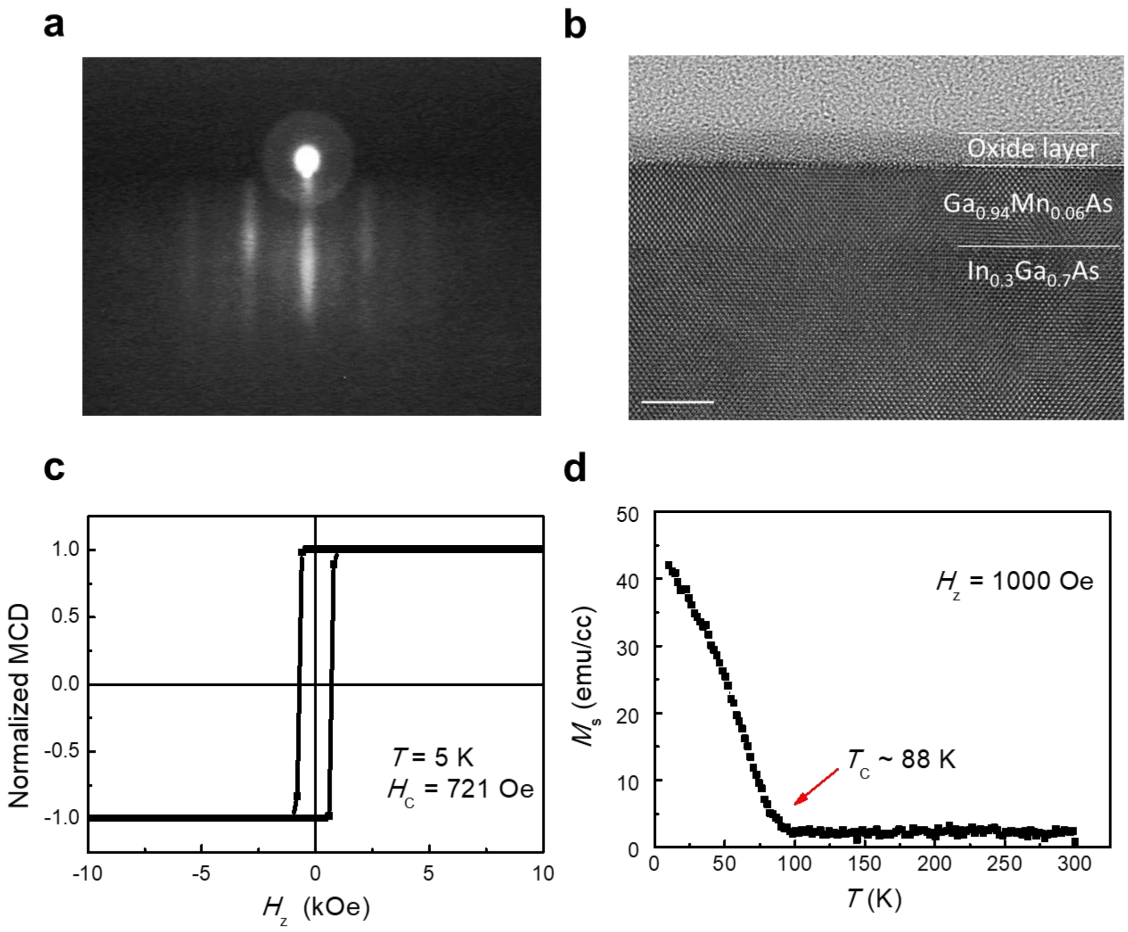

Supplementary Figure 1 | Fundamental properties of the $Ga_{0.94}Mn_{0.06}As$ thin film. a, RHEED pattern of the $Ga_{0.94}Mn_{0.06}As$ layer with the electron-beam azimuth along the $[\bar{1}10]$ direction. **b,** Transmission electron microscopy (TEM) lattice image of the $Ga_{0.94}Mn_{0.06}As/In_{0.3}Ga_{0.7}As$ thin film observed from the $[\bar{1}10]$ direction. The length of the scale bar is 5 nm. **c,** Perpendicular-magnetic-field H_z dependence of the magnetic circular dichroism (MCD) at 5 K. **d,** Temperature dependence of the saturation magnetization M_s with H_z = 1000 Oe. (Source data are provided as a Source Data file.)

Supplementary Note 2. Anomalous and planar Hall effects and anisotropy field of the $Ga_{0.94}Mn_{0.06}As$ at 40 K

Supplementary Figure 2a shows the Hall resistance R_H, which is dominated by the anomalous Hall effect (AHE), of the $Ga_{0.94}Mn_{0.06}As$ thin film at 40 K. From the result, R_H is changed between around ±1.7 kΩ during the magnetization reversal between the +*z* and –*z* directions. In addition, the value of the anisotropy field (H_{an}) of this sample at 40 K is estimated by the result of the in-plane magnetic-field dependence of the R_H as plotted in Supplementary Fig. 2b. With the comparison of the coercivity (H_C) in Supplementary Fig. 2a (H_C = 156 Oe) and Supplementary Fig. 2b (H_C = 2300 Oe), there is a 3.9° misalignment of the magnetic field due to the misalignment of the magnet and/or the sample setting, which results in an additional AHE signal. Based on the Stoner-Wohlfarth model, H_{an} and H_y are related by $H_{an}\sin(2\varphi) = H_y\sin(\varphi - \Delta\theta)$, where φ is the angle between the magnetization and the *y* axis and $\Delta\theta$ (~ 3.9°) is the misalignment angle. Using H_y = 4.2 kOe and sin φ = 0.5, where R_H is half of the maximum value, 1.7 kΩ, H_{an} ~ 2.13 kOe is estimated. Supplementary Figure 2c shows the planer Hall resistance (R_{PHE}) measured at 40 K with a current of ± 0.01mA applied along the $[\bar{1}10]$ direction and a fixed strong magnetic field of 10 kOe applied at an angle θ from the $[\bar{1}10]$ direction in the *x*-*y* plane, in which the magnetization is aligned along the magnetic-field direction. Here, the illustrations of the current (*J*), the external magnetic field (10 kOe) and θ are described as shown in Supplementary Fig. 2d. In Supplementary Fig. 2c, we set the scale of R_{PHE} from –1.5 kΩ to +1.5 kΩ, which is the same as that shown in Fig. 3b of the main manuscript (SOT switching curves at various temperatures). From the result, we can conclude that the planar Hall effect (PHE) is negligibly small in our system.

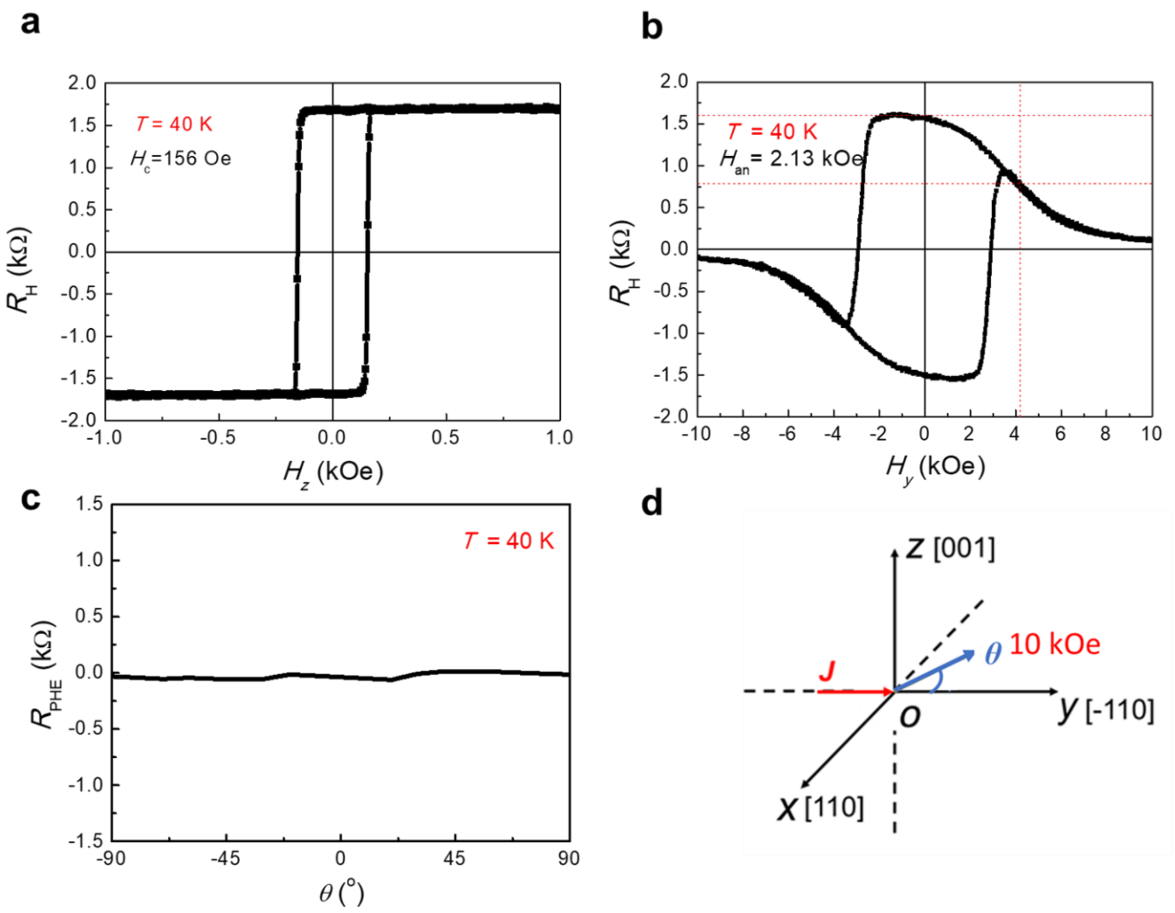

Supplementary Figure 2 | Anomalous and planar Hall effects and anisotropy field. a, Hall resistance R_H, which is dominated by the anomalous Hall effect (AHE), of $Ga_{0.94}Mn_{0.06}As$ at 40 K. **b,** In-plane magnetic-field H_y dependence of R_H. **c,** R_{PHE} measured with a current of ± 0.01 mA and an external field of 10 kOe applied at an angle θ from the $[\bar{1}10]$ direction in the x-y plane at 40 K. **d,** Illustrations of the current (J), the external magnetic field (10 kOe) and θ. (Source data are provided as a Source Data file.)

Supplementary Note 3. Temperature dependence of magnetic anisotropy of the $Ga_{0.94}Mn_{0.06}As$ thin film

The temperature-dependent hysteresis curves of the $Ga_{0.94}Mn_{0.06}As$ thin film with magnetic fields applied along the [100], [110], [$\bar{1}$10], and [001] directions are shown in Supplementary Fig. 3, which indicates that the magnetization easy axis of the $Ga_{0.94}Mn_{0.06}As$ thin film is along the [001] axis and the anisotropy energy decreases with the increase of temperature.

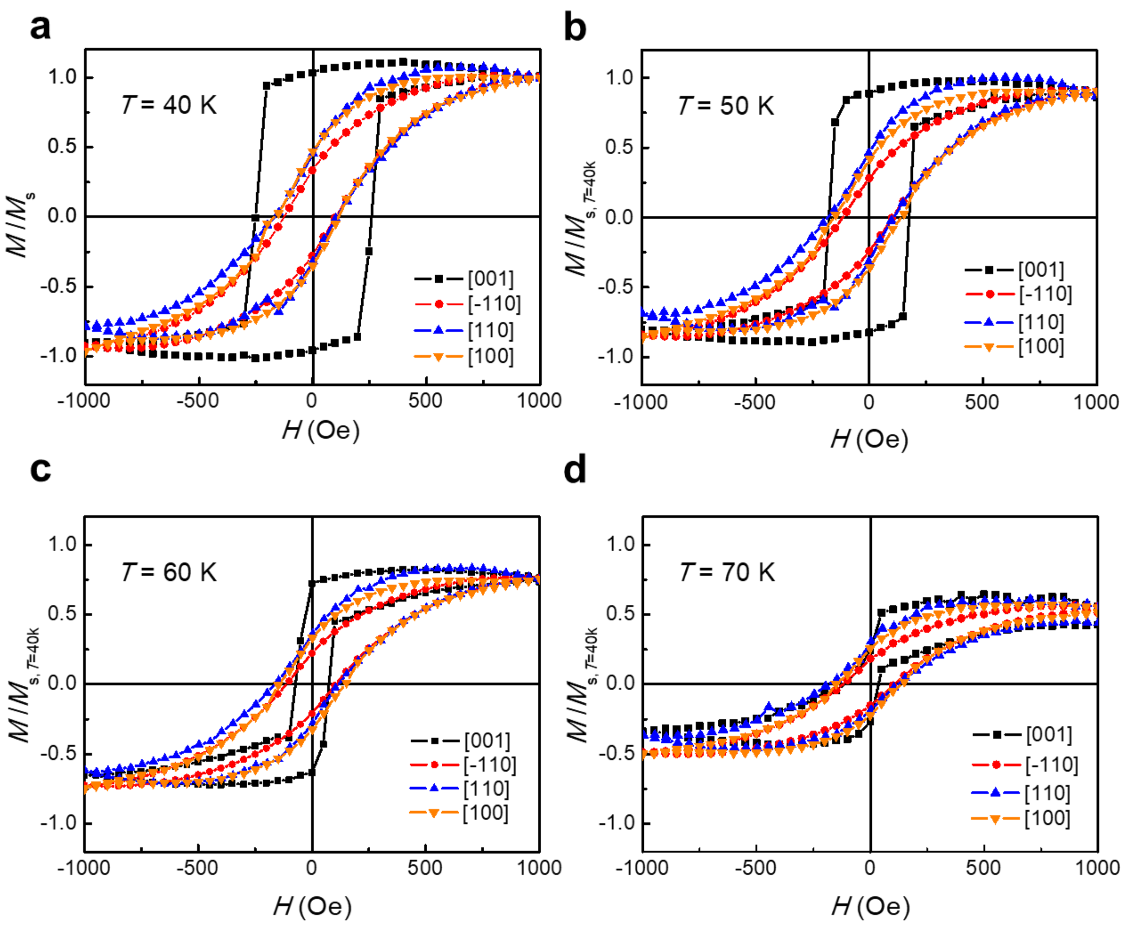

Supplementary Figure 3 | Temperature dependence of magnetization hysteresis. Magnetization hysteresis curves of the $Ga_{0.94}Mn_{0.06}As$ thin film measured with various magnetic field orientations at temperatures of **a,** 40 K, **b,** 50 K, **c,** 60 K and **d,** 70 K. (Source data are provided as a Source Data file.)

Supplementary Note 4. Solution of the Landau-Lifshitz-Gilbert equation

The Landau-Lifshitz-Gilbert (LLG) equation is expressed as

$$\dot{\hat{\mathbf{m}}} = -\gamma\, \hat{\mathbf{m}} \times \hat{\mathbf{H}} + \alpha\, \hat{\mathbf{m}} \times \dot{\hat{\mathbf{m}}} + \zeta_{\mathrm{DLT}}(\hat{\mathbf{m}} \times \hat{\boldsymbol{\sigma}}_x \times \hat{\mathbf{m}}) + \zeta_{\mathrm{FLT}}(\hat{\boldsymbol{\sigma}}_x \times \hat{\mathbf{m}}), \quad (1)$$

where $\hat{\mathbf{m}}$ represents the unit magnetization vector; $\dot{\hat{\mathbf{m}}}$ is the derivative of $\hat{\mathbf{m}}$ with respect to time; $\hat{\mathbf{H}}$ is the effective field consisting of the external field, the anisotropy field, Dresselhaus effective magnetic field H_{D} and Rashba effective magnetic field H_{R}; $\hat{\boldsymbol{\sigma}}_x$ is the x component of the spin polarization vector; γ is the gyromagnetic ratio; α is the damping constant; ζ_{DLT} is the damping-like torque (DLT) coefficient; and ζ_{FLT} is the field-like torque (FLT) coefficient. Then we can obtain $\hat{\mathbf{m}} \times \dot{\hat{\mathbf{m}}}$ as shown below.

$$\hat{\mathbf{m}} \times \dot{\hat{\mathbf{m}}} = -\gamma\, \hat{\mathbf{m}} \times \left(\hat{\mathbf{m}} \times \hat{\mathbf{H}}\right) + \alpha\, \hat{\mathbf{m}} \times \left(\hat{\mathbf{m}} \times \dot{\hat{\mathbf{m}}}\right) + \zeta_{\mathrm{DLT}}\hat{\mathbf{m}} \times (\hat{\mathbf{m}} \times \hat{\boldsymbol{\sigma}}_x \times \hat{\mathbf{m}}) + \zeta_{\mathrm{FLT}}\hat{\mathbf{m}} \times (\hat{\boldsymbol{\sigma}}_x \times \hat{\mathbf{m}})$$

$$= -\gamma\, \hat{\mathbf{m}} \times \hat{\mathbf{m}} \times \left(\hat{\mathbf{H}} + \frac{\zeta_{\mathrm{FLT}}}{\gamma}\hat{\boldsymbol{\sigma}}_x\right) - \alpha\, \dot{\hat{\mathbf{m}}} + \zeta_{\mathrm{DLT}}\hat{\mathbf{m}} \times \hat{\boldsymbol{\sigma}}_x. \quad (2)$$

By Replacing $\hat{\mathbf{m}} \times \dot{\hat{\mathbf{m}}}$ in Supplementary Eq. (1) with Supplementary Eq. (2), we obtain

$$\dot{\hat{\mathbf{m}}} = -\gamma\, \hat{\mathbf{m}} \times \hat{\mathbf{H}} + \alpha \left[-\gamma\, \hat{\mathbf{m}} \times \hat{\mathbf{m}} \times \left(\hat{\mathbf{H}} + \frac{\zeta_{\mathrm{FLT}}}{\gamma}\hat{\boldsymbol{\sigma}}_x\right) - \alpha\, \dot{\hat{\mathbf{m}}} + \zeta_{\mathrm{DLT}}\hat{\mathbf{m}} \times \hat{\boldsymbol{\sigma}}_x\right] + \zeta_{\mathrm{DLT}}(\hat{\mathbf{m}} \times \hat{\boldsymbol{\sigma}}_x \times \hat{\mathbf{m}}) +$$
$$\zeta_{\mathrm{FLT}}(\hat{\boldsymbol{\sigma}}_x \times \hat{\mathbf{m}}).$$

By moving $\frac{(1+\alpha^2)\dot{\hat{\mathbf{m}}}}{\gamma}$ to the left side of the equation, Supplementary Eq. (3) is obtained.

$$\frac{(1+\alpha^2)\dot{\hat{\mathbf{m}}}}{\gamma} = -\,\hat{\mathbf{m}} \times \hat{\mathbf{H}} + \left(\frac{\zeta_{\mathrm{FLT}} - \alpha\,\zeta_{\mathrm{DLT}}}{\gamma}\right)(\hat{\boldsymbol{\sigma}}_x \times \hat{\mathbf{m}}) - \alpha\hat{\mathbf{m}} \times \hat{\mathbf{m}} \times \hat{\mathbf{H}} - \left(\frac{\alpha\,\zeta_{\mathrm{FLT}} + \zeta_{\mathrm{DLT}}}{\gamma}\right)\hat{\mathbf{m}} \times \hat{\mathbf{m}} \times \hat{\boldsymbol{\sigma}}_x$$

$$= -\hat{\mathbf{m}} \times \left[\hat{\mathbf{H}} + \left(\frac{\zeta_{\mathrm{FLT}} - \alpha\,\zeta_{\mathrm{DLT}}}{\gamma}\right)\hat{\boldsymbol{\sigma}}_x\right] - \alpha\hat{\mathbf{m}} \times \hat{\mathbf{m}} \times \left[\hat{\mathbf{H}} + \left(\frac{\alpha\,\zeta_{\mathrm{FLT}} + \zeta_{\mathrm{DLT}}}{\alpha\gamma}\right)\hat{\boldsymbol{\sigma}}_x\right]. \quad (3)$$

Here, we replace $\zeta_{\mathrm{DLT}}\,\hat{\boldsymbol{\sigma}}_x$ with $r\gamma\,\hat{\mathbf{S}}_x$ and $\zeta_{\mathrm{FLT}}\,\hat{\boldsymbol{\sigma}}_x$ with $\frac{\gamma(1-r)}{\alpha}\hat{\mathbf{S}}_x$, where $\hat{\mathbf{S}}_x$ is the effective magnetic field in the x direction and r expresses the strength of the DLT relative to the total SOT: when r

is 0, only the FLT is present, and when r is 1, only the DLT is present. Thus, Supplementary Eq. (3) can be expressed as

$$\frac{\dot{\hat{\mathbf{m}}}}{\gamma'} = -\hat{\mathbf{m}} \times \left(\hat{\mathbf{H}} + \beta \hat{\mathbf{S}}_x\right) - \alpha\, \hat{\mathbf{m}} \times \hat{\mathbf{m}} \times \left(\hat{\mathbf{H}} + \frac{1}{\alpha} \hat{\mathbf{S}}_x\right), \tag{4}$$

where

$$\gamma' = \frac{\gamma}{1+\alpha^2}\,, \beta = \frac{1-r(1+\alpha^2)}{\alpha}.$$

Then, we introduce $\hat{\mathbf{m}} = \begin{pmatrix} m_x \\ m_y \\ m_z \end{pmatrix}$, $\hat{\mathbf{S}}_x = \begin{pmatrix} S_x \\ 0 \\ 0 \end{pmatrix}$, $\hat{\mathbf{H}} = \begin{pmatrix} 0 \\ H_y \\ H_{\mathrm{an}} m_z \end{pmatrix}$ into Supplementary Eq. (4), and the LLG equation can be solved as

$$\begin{pmatrix} \Delta m_x \\ \Delta m_y \\ \Delta m_z \end{pmatrix} = \Delta t' \left[\begin{pmatrix} H_{\mathrm{an}} m_y m_z - H_y m_z \\ \beta S_x m_z - H_{\mathrm{an}} m_x m_z \\ H_y m_x - \beta S_x m_y \end{pmatrix} + \left(S_x m_x + \alpha H_y m_y + \alpha H_{\mathrm{an}} m_z m_z\right) \begin{pmatrix} m_x \\ m_y \\ m_z \end{pmatrix} - \begin{pmatrix} S_x \\ \alpha H_y \\ \alpha H_{\mathrm{an}} m_z \end{pmatrix} \right],$$

where

$$\Delta t' = -\gamma' \Delta t = -\frac{\gamma}{1+\alpha^2} \Delta t\,.$$
