# Supplementary material for: Efficient full spin–orbit torque switching in a single layer of a perpendicularly magnetized single-crystalline ferromagnet
Source: Nat Commun. 2019 Jun 13;10:2590. doi: 10.1038/s41467-019-10553-x (PMC6565668; doi:10.1038/s41467-019-10553-x)
Supplement: Supplementary file 2 — Peer Review File [file 41467_2019_10553_MOESM2_ESM.pdf]

Editorial Note: This manuscript has been previously reviewed at another journal that is not operating a transparent peer review scheme. This document only contains reviewer comments and rebuttal letters for versions considered at Nature Communications .

Reviewers' comments:

Reviewer #1 (Remarks to the Author):

To the extent that my previous report contained criticisms, I think the authors have adequately addressed them. I also believe that the authors have adequately addressed the criticisms by the other referees. Here are my responses to the new set of questions we are asked to consider.

What are the major claims of the paper? Are they novel and will they be of interest to others in the community and the wider field?

The authors show the first demonstration of robust spin-orbit-torque-induced magnetization reversal in a single film. Other authors have observed switching but not reversal and others have measured the torque responsible for the switching in this system. However, I believe that the present results will be of interest to others in the community because magnetization reversal is the ultimate goal for these studies. Due to the low transition temperature of GaMnAs, it is unlikely that will be commercially important in the near future, but it is a well studied system and the present work adds to that knowledge base.

Is the work convincing, and if not, what further evidence would be required to strengthen the conclusions?

I find the work convincing and believe the authors adequately addressed the criticisms raised by the other referees.

On a more subjective note, do you feel that the paper will influence thinking in the field?

While I do not believe there are substantially new ideas per se in this paper, I do believe that the result it reports will influence the field because it shows that an effect that is plausible in fact exists. I think there is significant value in experimental demonstrations even if there is nothing surprising in the results. This result will encourage workers in the field to try to identify ferromagnetic materials with high transition temperature and which break inversion symmetry.

I do not have any further questions and concerns about the paper.

Reviewer #2 (Remarks to the Author):

My overall impression is that the authors have provided more important experimental results in the Supplementary Information, while the main manuscript still only has the original three figures. In order to be considered for publication in Nature Communications or other Nature journals, the manuscript usually needs to be complete and the most important information should be in the main manuscript, not in the Supplementary Information. The switching phase diagram and its temperature dependence (the main results in Fig. S4 and Fig. R4) are important, so they should be included in the main manuscript. Also, the calibration of the spin-orbit torque strength (Fig. S5 and Fig. S6) is important for knowing the property of this new perpendicular magnetic semiconductor, and this information should be included in the main manuscript. I am satisfied with most of the responses, but these new experimental results should be added in the main manuscript in order to provide the readers a complete and comprehensive picture of this whole work.

Reviewer #3 (Remarks to the Author):

The manuscript has been revised according to all three reviewer's comments. Most of the comments have been responded carefully. However, there are still a couple issues the authors should clarify before the paper can be published in Nature Communication.

1. Regarding the anisotropy field used in the simulation and analysis, its value is only attained from Fig. S2 at T = 40 K. The value is 2.13 kOe, which seems not consistent with the SQUID data in Fig. S3, which show in-plane M is approaching saturation at 1000 kOe at 40 K. The author should conduct more careful measurement to obtain the anisotropy field and its temperature dependence. Since Joule heating effect (~10K claimed by the authors) should be also considered in the simulation/fitting, a temperature (or current) dependent anisotropy field should be measured and considered in the calculation. In this way, the obtained value of r should be revised accordingly.

2. Regarding the planar Hall effect, it is measured as M away from the current direction (y) with sin(2theta). The Fig. S3 data did not provide the information about this , without this information, the obtained value of r from simulation is not solid. Note that, the data in Fig. 2 is measured with field along current direction, PHE is not present. PHE data is occurring when M along 45 degree away from current direction. So it is expected to be present around the switching point according to Fig. 3.

3. Some data (such as critical current phase diagram etc.) in supplementary and response letter should be included in the main text, in order to enhance the paper presentation.

Response Letter

Firstly, we would like to thank all the reviewers for careful reading of our manuscript (NCOMMS-19-01168-T) and previous Response Letter, and for his/her valuable comments that helped us improve the quality of our manuscript. Here, we describe our responses to all the criticisms of the reviewers (shown as blue characters in this Response Letter) and summarize how we revised our manuscript. The revised parts are shown in red in this Response Letter, revised main manuscript and Supplementary Information.

Reviewer #2 (Remarks to the Author):

My overall impression is that the authors have provided more important experimental results in the Supplementary Information, while the main manuscript still only has the original three figures. In order to be considered for publication in Nature Communications or other Nature journals, the manuscript usually needs to be complete and the most important information should be in the main manuscript, not in the Supplementary Information. The switching phase diagram and its temperature dependence (the main results in Fig. S4 and Fig. R4) are important, so they should be included in the main manuscript. Also, the calibration of the spin-orbit torque strength (Fig. S5 and Fig. S6) is important for knowing the property of this new perpendicular magnetic semiconductor, and this information should be included in the main manuscript. I am satisfied with most of the responses, but these new experimental results should be added in the main manuscript in order to provide the readers a complete and comprehensive picture of this whole work.

We would like to thank the reviewer for the comment. Following the comment, we added the description on the estimation of the heating effect, switching phase diagram, and characterization of the spin-orbit torque from page 7 to line 9 on page 9 in the revised main manuscript. The corresponding figures are inserted as Fig. 3 on page 19 and Fig. 4 on page 20 in the revised main manuscript.

Revised parts:
We added new sections "**Estimation of the heating effect and switching phase diagram**" in page 7-8 and "**Spin-orbit torque strength in the $Ga_{0.94}Mn_{0.06}As$ thin film**" in page 8-9 in the revised main manuscript. Fig. 3 and Fig. 4 are added in the revised main manuscript in page 19 - 20.

Reviewer #3 (Remarks to the Author):

1. Regarding the anisotropy field used in the simulation and analysis, its value is only attained from Fig. S2 at T = 40 K. The value is 2.13 kOe, which seems not consistent with the SQUID data in Fig. S3, which show in-plane M is approaching saturation at 1000 Oe at 40 K. The author should conduct more careful measurement to obtain the anisotropy field and its temperature dependence. Since Joule heating effect (~10K claimed by the authors) should be also considered in the simulation/fitting, a temperature (or current) dependent anisotropy field should be measured and considered in the calculation. In this way, the obtained value of r should be revised accordingly.

We would like to thank the reviewer for this comment. Firstly, to suppress the Joule heating effect in the simulation/fitting, in the revised main manuscript, we fitted the experimental result measured at 40 K for the device *capped with metal electrodes as heat sinks*, in which the heating effect is much reduced in comparison with the device with no capping layer used in the previous manuscript (see Fig. 3a in the revised main manuscript). The new fitting result is shown in Fig. R1b (= Fig. 5b in the revised main manuscript), in which the experimental result can still be well fitted with the calculation result when the ratio r of the damping-like torque (DLT) relative to the total spin-orbit torque (SOT) is 0.95, indicating that both the DLT and field-like torque (FLT) act on SOT switching but that the DLT is dominant. This is exactly the same result as that in our previous manuscript. Following this result, we changed the sentence "Here, we consider the case of Fig. 2a (*i.e., J // y* with H_y)" (line 4 and 5 on page 7 in the previous main manuscript) to "Here, we consider the SOT switching result obtained at 40 K for the device with metal electrodes as heat sinks (see Fig. 3b) (*i.e., J // y* with H_y). " at line 13 and 14 on page 9 and replaced Fig. 5b with Fig. R1b on page 21 in the revised main manuscript.

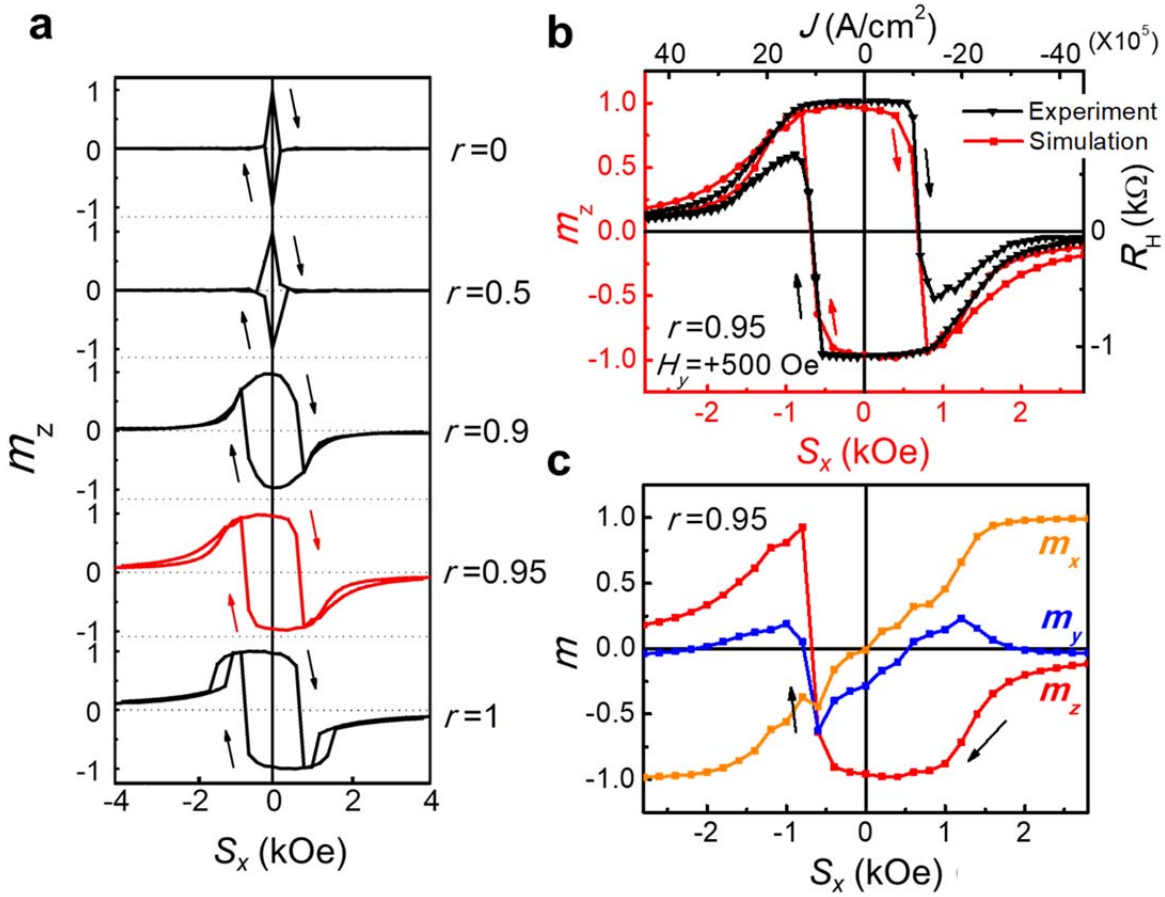

Figure R1 (= Fig. 5 in the revised main manuscript) | Landau-Lifshitz-Gilbert (LLG) simulation results and comparison with experimental results. a, Simulated SOT switching with different r values of 0, 0.5, 0.9, 0.95 and 1. The parameters used here are H_y = 500 Oe, H_{an} = 2.13 kOe, and α = 0.05. **b,** Simulated SOT switching at r = 0.95 and experimental results with H_y = 500 Oe. Note that the direction of the J axis is reversed because the signs of J and S_x are opposite [*e.g.,* when $J > 0$, S_x (or σ_x) < 0, as shown in Fig. 2c]. **c,** Calculated values of m_x, m_y and m_z as functions of S_x.

Secondly, the difference in the anisotropy field (H_{an}) between supplementary Fig. 2 and supplementary Fig. 3 (in the revised Supplementary Information (SI)) clearly originates from the size effect resulting from the device fabrication process. Supplementary Fig. 3 shows the hysteresis curves measured by SQUID on the mm-scale sample at various temperatures, while the transport measurement result shown in supplementary Fig. 2 was measured on the patterned cross-bar device with a width of 5 μm. Because we use the same cross-bar device for the SOT switching measurements, our estimation of the anisotropy field is reasonable for understanding the SOT switching. In addition, we conducted the measurement of H_{an} again at different temperatures in the cross-bar device capped with metal electrodes, and the results are shown as the black curve in Fig. R2, from which we can conclude that H_{an} decreases with the increase in the temperature. Comparing the value of H_{an} at 40 K shown in Fig. R2 with the previous H_{an} measured in the device with no capping metal electrodes in last April (shown in the previous manuscript), H_{an} increases from the previous value of 2.13 kOe to 3.73 kOe, which is probably caused by the oxidization of the sample surface as the time goes by. The coercivity (H_C) also increases from 156 Oe to 318 Oe at 40 K, and the ratio of H_C and H_{an} almost keeps constant.

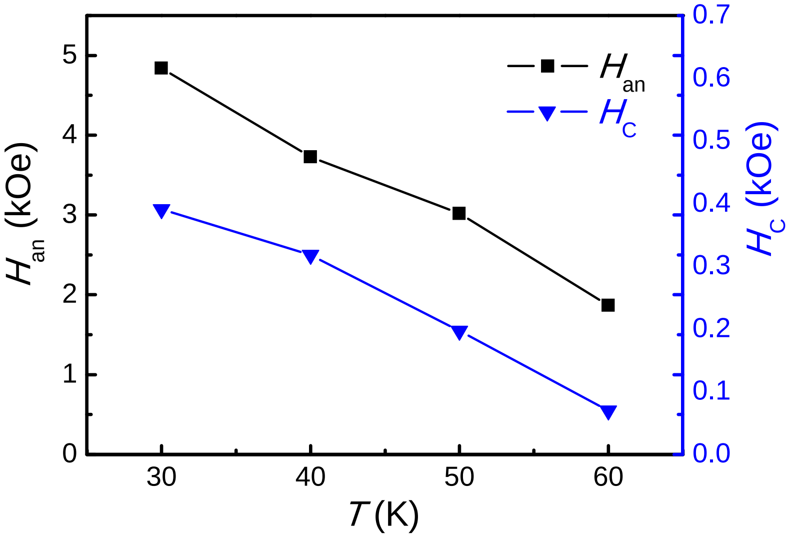

Figure R2 | Temperature dependence of the anisotropy field H_{an} (black curve) and the coercivity H_C (blue curve) in the $Ga_{0.94}Mn_{0.06}As$ cross-bar capped with metal electrodes.

Thirdly, while the r value expresses the ratio of DLT and FLT, what the anisotropy field influences here is the critical switching current density. Figure R3 shows the simulation results with the same parameters as those used in the revised manuscript except H_{an} = 4.2 kOe (on the other hand, H_{an} = 2.13 kOe in our revised main manuscript), indicating that the shapes of the simulation magnetization switching curves are almost same as that shown in Fig. 5 of the revised manuscript, although the critical switching effective magnetic field (S_x) becomes larger. From the fitting result shown in Fig. R3b, the r value is estimated to be 0.95, which is the same as that in the revised main manuscript, and this means that the DLT is dominant during the SOT switching. Therefore, the obtained value r = 0.95 is solid and reliable.

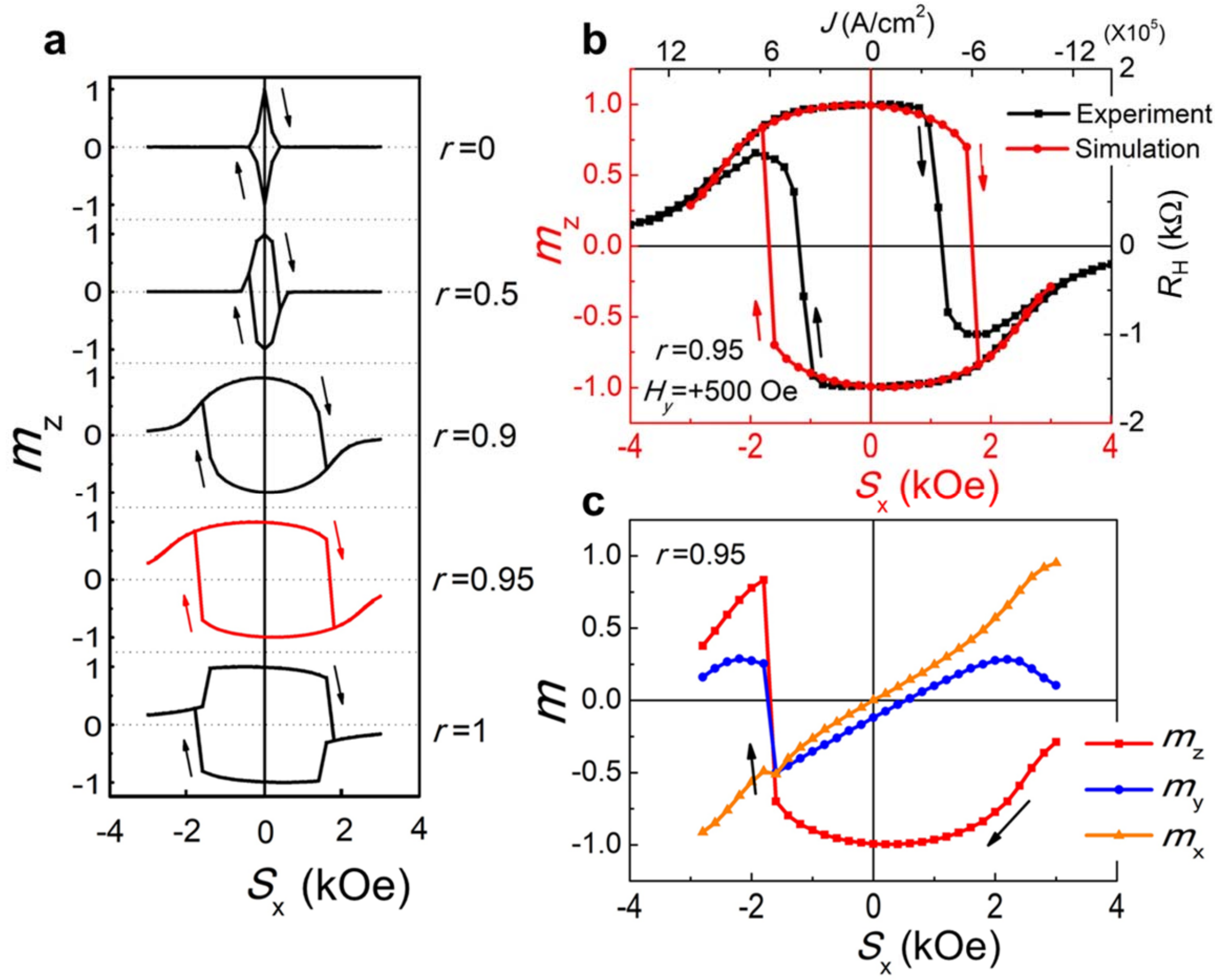

Figure R3 | LLG simulation results and comparison with experimental results. a, Simulated SOT switching with different r values of 0, 0.5, 0.9, 0.95 and 1. The parameters used here are H_y = 500 Oe, H_{an} = 4.2 kOe (on the other hand, we set H_{an} = 2.13 kOe in Fig. 5 in the revised main manuscript), and α = 0.05. **b,** Simulated SOT switching at r = 0.95 and experimental results with H_y = 500 Oe. Note that the direction of the J axis is reversed because the signs of J and S_x are opposite. **c,** Calculated values of m_x, m_y and m_z as functions of S_x. Both of the fitting results shown in Fig. 5 and in Fig. R3 indicate r = 0.95.

Revised parts:

We revised the sentence "Here, we consider the case of Fig. 2a (*i.e., J // y* with H_y)" (line 4 and 5 on page 7 in the previous main manuscript) to "Here, we consider the SOT switching result obtained at 40 K for the device with metal electrodes as heat sinks (see Fig. 3b) (*i.e., J // y* with H_y). " at line 13 and 14 on page 9, and replaced Fig. 5b with Fig. R1b on page 21 in the revised main manuscript.

2. Regarding the planar Hall effect, it is measured as M away from the current direction (y) with sin(2theta). The Fig. S3 data did not provide the information about this, without this information, the obtained value of r from simulation is not solid. Note that, the data in Fig. 2 is measured with field along current direction, PHE is not present. PHE data is occurring when M along 45 degree away from current direction. So it is expected to be present around the switching point according to Fig. 3.

We would like to thank the reviewer for the comment. In order to check whether the planar Hall Effect (PHE) is present *or not* in our work, we measured planar Hall resistance (R_{PHE}) at 40 K with a current of ± 0.01mA applied along the [$\bar{1}$10] direction and a fixed strong magnetic field of 10 kOe applied at an angle θ from the [$\bar{1}$10] direction in the *x*-*y* plane as shown in Fig. R4, in which the magnetization is aligned along the magnetic-field direction. Here, we set the scale of R_{PHE} in Fig. R4a from -1.5 kΩ to +1.5 kΩ, which is the same as that shown in Fig. 3b of the revised main manuscript (SOT switching curves at various temperatures). From the result, we can conclude that there is no obvious PHE in our system.

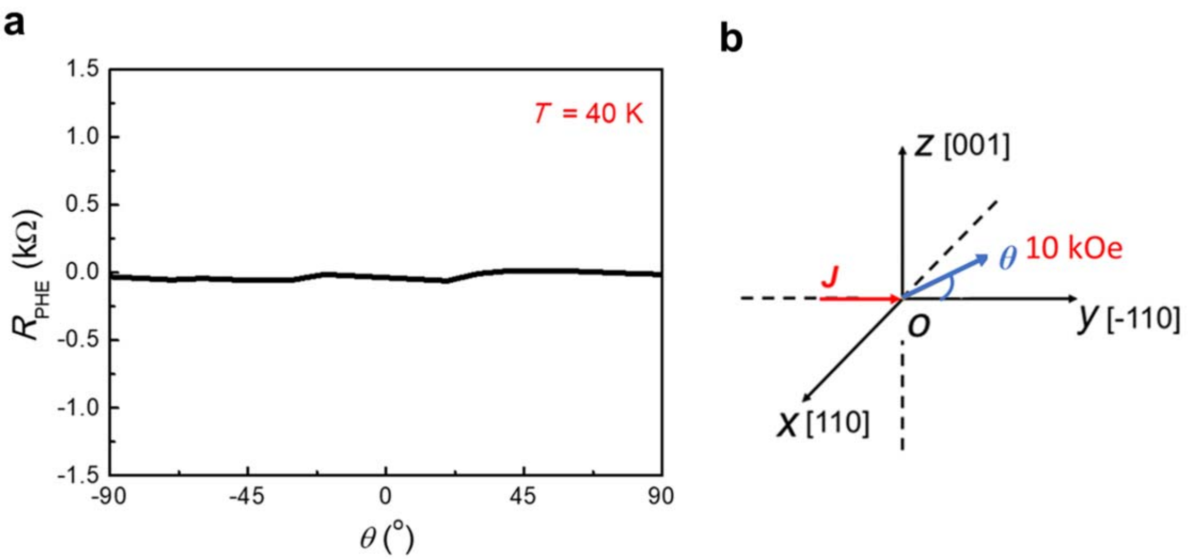

Figure R4 (= supplementary Fig. 2c,d in the revised Supplementary Information)| | a, R_{PHE} measured with a current of ± 0.01 mA and an external field of 10 kOe applied at an angle θ from the [$\bar{1}$10] direction in the *x*-*y* plane at 40 K. **b,** Illustrations of the current (*J*), the external magnetic field (10 kOe) and θ.

Revised parts:
We added the following sentences to the revised Supplementary Information (SI) from line 13 to line 22 on page 4 and added Fig. R4a,b as supplementary Fig. 2c,d on page 5 in SI :
"Supplementary Fig. 2c shows the planer Hall resistance (R_{PHE}) measured at 40 K with a current of ± 0.01mA applied along the [$\bar{1}$10] direction and a fixed strong magnetic field of 10 kOe applied at an angle θ from the [$\bar{1}$10] direction in the *x*-*y* plane, in which the magnetization is aligned along the magnetic-field direction. Here, the illustrations of the current (*J*), the external magnetic field (10 kOe) and θ are described as shown in supplementary Fig. 2d. In supplementary Fig. 2c, we set the scale of R_{PHE} from -1.5 kΩ to +1.5 kΩ, which is the same as

that shown in Fig. 3b of the main manuscript (SOT switching curves at various temperatures). From the result, we can conclude that the planar Hall effect (PHE) is negligibly small in our system."

3. Some data (such as critical current phase diagram etc.) in supplementary and response letter should be included in the main text, in order to enhance the paper presentation.

We would like to thank the reviewer for this comment. As shown in the response to the comment of Reviewer #2, some necessary data have been included in the main manuscript.

REVIEWERS' COMMENTS:

Reviewer #3 (Remarks to the Author):

The authors have responded all comments from both reviewers. I am satisfied with the response and the manuscript is now in a good form for the publication in Nature Communications.

Response Letter

We would like to thank all the reviewers for careful reading of our revised manuscript (NCOMMS-19-01168-A) and the satisfaction with the Response Letter.

Reviewer #3 (Remarks to the Author):

The authors have responded all comments from both reviewers. I am satisfied with the response and the manuscript is now in a good form for the publication in Nature Communications.
